# Usefulness of Acoustic Monitoring of Respiratory Rate in Patients Undergoing Endoscopic Submucosal Dissection

**DOI:** 10.1155/2016/2964581

**Published:** 2015-12-27

**Authors:** Takayoshi Suzuki, Shingo Tsuda, Hirohiko Nakae, Jin Imai, Kana Sawamoto, Maiko Kijima, Yoko Tsukune, Tetsufumi Uchida, Muneki Igarashi, Jun Koike, Masashi Matsushima, Toshiyasu Suzuki, Tetsuya Mine

**Affiliations:** ^1^Division of Gastroenterology and Hepatology, Department of Internal Medicine, Tokai University School of Medicine, 143 Shimokasuya, Isehara, Kanagawa 259-1193, Japan; ^2^Tokai University Tokyo Hospital, 1-2-5 Yoyogi, Shibuya-ku, Tokyo 151-0053, Japan; ^3^Department of Anesthesiology, Tokai University School of Medicine, 143 Shimokasuya, Isehara, Kanagawa 259-1193, Japan

## Abstract

*Aim*. The study assessed the usefulness of a recently developed method for respiratory rate (RR) monitoring in patients undergoing endoscopic submucosal dissection (ESD) under deep sedation. *Methods*. Study subjects comprised 182 consecutive patients with esophageal cancer or gastric cancer undergoing ESD. The usefulness of acoustic RR monitoring was assessed by retrospectively reviewing the patients' records for age, gender, height, weight, past history, serum creatinine, RR before ESD, and total dose of sedative. *Results*. Respiratory suppression was present in 37.9% of (69/182) patients. Continuous monitoring of RR led to detection of respiratory suppression in all these patients. RR alone was decreased in 24 patients, whereas both RR and blood oxygen saturation were decreased in 45 patients. Univariate analysis showed female gender, height, weight, and RR before treatment to be significantly associated with respiratory suppression. Multivariate analysis showed RR before treatment to be the only significant independent predictor [odds ratio (OR) 0.83, 95% confidence interval (CI) 0.73–0.95, and *P* = 0.006] of respiratory suppression. *Conclusion*. In this study, the difference in RR before treatment between patients with and without respiratory suppression was subtle. Therefore, we suggest that acoustic RR monitoring should be considered in patients undergoing ESD under sedation to prevent serious respiratory complications.

## 1. Introduction

Gastric cancer remains one of the most common causes of cancer death in the world [1]. The technique of endoscopic submucosal dissection (ESD) was initially developed in Japan. This technique is increasingly used around the world as it results in increased en bloc and complete resection rates and reduced local recurrence rate [2, 3]. Since this technique requires relatively long procedure time and involves complicated maneuvers, appropriate sedation to reduce patient's discomfort, anxiety, and pain is vital to achieve safe and effective ESD. However, sedation might lead to many serious unexpected events, such as apnea, hypotension, bradycardia, and cardiac arrest. Notably, these cardiorespiratory-related complications are a leading cause of morbidity and mortality during endoscopic procedures. Continuous venous access should be maintained to administer antagonists or resuscitated medications until sedated patients recover completely. Additionally, continuous monitoring of patient under sedation is essential for avoiding serious complications and ensuring safe endoscopic treatment. Standard monitoring of the patient undergoing endoscopic treatment includes pulse rate, arterial oxygen saturation, blood pressure, level of consciousness based on patient's ability to respond to verbal commands, and electrocardiography in case of selected high-risk patients with heart diseases, including cardiac arrhythmias. However, at present, there is no consensus on what kind of monitoring devices is needed during ESD.

It seems obvious that monitoring arterial oxygen saturation alone is not enough to detect hypoventilation or apnea as an early indicator of respiratory distress especially in patients receiving supplemental oxygen [4]. Therefore, the question of how to effectively monitor patient's respiratory rate (RR) during ESD under deep sedation is extremely crucial.

**Figure 1:**
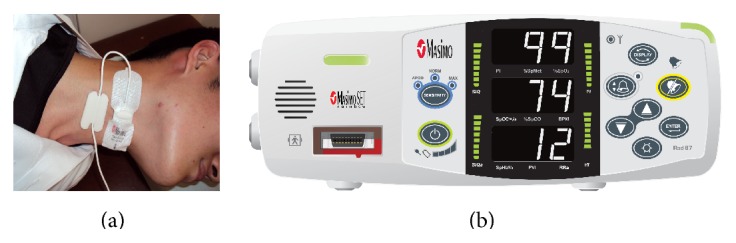
Acoustic respiratory rate monitoring device. (a) Monitoring sensor placed on the neck. (b) The display (Masimo Rad-87).

Acoustic RR monitoring (Masimo Rad-87) was recently developed by the Masimo Corporation. Patients were connected to a pulse oximeter with acoustic monitoring sensor. The sensor was applied to the patient's neck to detect respiratory vibrations originating in the walls of the large airways during breathing (Figure 1). Thereafter, those acoustic sounds transform into electrical signals that are converted to numerical measurements of RR. Recent studies have reported that acoustic RR monitoring is accurate and has sensitivity and specificity similar to those of capnometry in extubated patients after general anesthesia [5]. However, the usefulness of this new noninvasive technique for monitoring the RR has not been investigated in patients undergoing endoscopic treatment under sedation.

In this background, this study evaluates the risk factors for respiratory suppression in patients undergoing ESD under deep sedation and assesses the usefulness of acoustic RR monitoring in these patients.

## 2. Methods 

This study conducted between September 2012 and March 2014 at Tokai University Hospital comprised of 182 consecutive patients with esophageal cancer or gastric cancer undergoing ESD. Continuous acoustic RR monitoring was done in all these patients. Initially, patients received 1-2 mg midazolam and 35 mg pethidine hydrochloride for induction of venous anesthesia. Additional midazolam (1-2 mg) was repeatedly administered to maintain the depth of sedation. An analgesic (15 mg pentazocine) was given intravenously in case patients exhibited pain with movement. The level of sedation in each patient was assessed using the American Society of Anesthesiologists classification and maintained at a moderate to deep level.

All ESD procedures were performed using Olympus video endoscopes (Olympus Optical Co., Ltd., Tokyo, Japan) with a transparent cap attached at the tip of the scope. Mainly, IT knife 2 and ClutchCutter were used in ESD in patients with early gastric cancer and esophageal cancer, respectively. Pulse rate, arterial oxygen saturation, blood pressure (BP), level of consciousness, and RR were monitored during ESD. Electrocardiography monitoring was performed in selected high-risk patients with heart diseases. Oxygen was administered using a nasal cannula at a flow rate of 2 L/min. In case of severe cardiopulmonary suppression, an antagonist of midazolam was administered to the patients.

We identified the risk factors for respiratory suppression in patients undergoing ESD under sedation and assessed the usefulness of recently developed method of acoustic RR monitoring in these patients. Univariate and multivariate regression analyses were performed to identify the risk factors that led to respiratory suppression during ESD under sedation. We hypothesized that the probable factors associated with respiratory suppression include gender, age, height, weight, past history, serum creatinine (Cr), RR before treatment, circulatory suppression, total dose of sedative (midazolam), procedure time, and type of ESD. Then, patients' records were retrospectively reviewed for those factors.

Respiratory suppression was defined as a decrease in blood oxygen saturation of ≤91% for more than 10 seconds, or a decrease in the RR of ≤8/min for more than 10 seconds [6, 7]. Circulatory suppression was defined as a decrease in systolic BP to less than 90 mmHg. A chin lift or jaw thrust maneuver with increasing the oxygen supplementation was used in case of respiratory suppression. Moreover, further midazolam doses were withheld until recovery of respiratory condition.

This study was approved by the Ethics Review Board of Tokai University School of Medicine. All the study participants provided informed written consent prior to the procedure.

### 2.1. Statistical Analyses

Continuous variables were expressed as mean ± standard deviation (SD). To elucidate risk factors for respiratory suppression, univariate analysis was performed using the Chi-square test and the Mann-Whitney *U* test. Significant univariate risk factors were used to build a multivariate logistic regression model in order to identify independent risk factors for respiratory suppression. Statistical analyses were performed using the StatFlex (version 6.0) computer program. A *P* value of < 0.05 was considered statistically significant.

## 3. Results

The study involved retrospective analysis of prospectively collected data on 182 consecutive patients including 24 patients with esophageal cancer and 158 patients with early gastric cancer. The incidence of respiratory suppression was 37.9% (69/182). RR alone was decreased in 24 patients, whereas both RR and blood oxygen saturation were decreased in 45 patients. There was no patient with decreased blood oxygen saturation alone. The incidence of circulatory suppression was 25.8%. Although some patients developed transient cardiorespiratory suppression, no severe complications attributable to sedation were reported during ESD. Therefore, resuscitation maneuvers or resuscitation drugs were not used in any patients with respiratory suppression. Similarly, no serious complications related to the ESDprocedure were noted.

As shown in Table 1, univariate analysis revealed that there were no significant differences in age, past history, serum creatinine, dose of sedative, procedure time, incidence of circulatory suppression, and type of ESD between the patients with and without respiratory suppression. Notably, univariate analysis showed that female gender, height, weight, and RR before treatment were significantly associated with respiratory suppression. To rule out confounding factors, a logistic regression analysis was performed (Table 2). The RR before treatment was the only independent risk factor for respiratory suppression during ESD under deep sedation.

## 4. Discussion

Although recent studies have reported that acoustic RR monitoring is well tolerated and has sensitivity and specificity similar to those of capnometry in patients after general anesthesia, there is little information about the usefulness of this monitoring technique in patients undergoing gastrointestinal endoscopic treatment under conscious sedation [5, 8]. To the best of our knowledge, this is the first study to evaluate the usefulness of acoustic RR monitoring in patients undergoing ESD. In fact, we used it to measure the incidence of respiratory suppression in patients undergoing ESD and to assess the risk factors for respiratory suppression in these patients. Previous studies have shown that the incidence of respiratory suppression in patients undergoing gastrointestinal endoscopic treatment under conscious sedation ranges from 10 to 70%, depending on the definition of respiratory suppression, patient population, type of endoscopy, and type of sedatives [9, 10]. Markedly, in these studies, pulse oximetrywas used as a surrogate measure of ventilation. However, it seems obvious that the physiological range (93%–98%) measured by pulse oximetry would not ensure adequate measurement of respiratory ventilation. Furthermore, this monitor lags far behind to detect respiratory suppression especially in patients receiving supplemental oxygen [4]. An important criterion in monitoring respiratory function during endoscopic treatment is the ability to find out those respiratory suppressed patients who remain undetected by pulse oximetry. Consequently, some recent studies recommend monitoring the patients undergoing gastrointestinal endoscopy under sedation for respiratory suppression using capnometry, since it has been increasingly acknowledged as a superior method for detecting early respiratory suppression during therapeutic upper endoscopy [9]. However, it is technically difficult to measure RR using capnometry in patients receiving nasal oxygen, as both these procedures usually require nasal cannulas or face masks. Furthermore, patients undergoing ESD could be breathing through the open mouth contributing to the unreliability of the measured RR. Additionally, since carbon dioxide insufflation is being increasingly used in ESD to reduce abdominal pain and discomfort caused by bowel hyperextension, it may be quite difficult to accurately monitor RR by measuring end tidal carbon dioxide using capnometry. Therefore, an accurate and reliable method of monitoring the RR in patients undergoing ESD is an urgent need.

In this study, we used acoustic RR monitoring continuously in patients undergoing ESD. Based on our definition of respiratory suppression, approximately 40% of the study subjects developed at least one episode of respiratory suppression. A decrease in both RR and blood oxygen saturation occurred approximately in two-thirds of the patients who developed respiratory suppression, whereas no patient with respiratory suppression had a decrease in blood oxygen saturation alone. Detailed analysis revealed that a decrease in RR alone occurred in 35% of the patients who developed respiratory suppression. In the absence of acoustic RR monitoring, respiratory suppression could not have been detected in these patients by measuring the blood oxygen saturation alone and, consequently, some of these patients might have progressed to clinical situations similar to apnea. Resuscitation maneuvers or resuscitation drugs were not used for any patients with respiratory suppression in this study. However, monitoring RR during ESD might be of great importance for avoiding overdose of sedative especially in patients with decrease in RR. Taken together, these data corroborate the indispensability of acoustic RR monitoring in patients undergoing ESD. Furthermore, this monitoring technique is easier to use and less expensive than capnography.

Published literature has revealed that longer duration of procedures [11], body weight [12–14], hypertension, diabetes, heart diseases, procedures that combine esophagogastroduodenoscopy and colonoscopy, and increasing age [15, 16] are risk factors for respiratory suppression in patients undergoing gastrointestinal endoscopy under sedation. However, these risk factors would depend on many other variables such as study design, patient population, type of endoscopy, and type of sedatives, to name a few. In this study, univariate analysis revealed that female gender, low height and weight, and low RR before treatment were significantly associated with respiratory suppression. Multivariate analysis revealed that RR before treatment was the only significant independent predictor of respiratory suppression during ESD under deep sedation. Notably, in our study, the difference in RR between patients with and without respiratory suppression was subtle. This underscores the practical difficulty in beforehand predicting the patients who would subsequently develop respiratory suppression during ESD. When the patients in whom respiratory suppression was detected solely by acoustic RR monitoring were compared with those who did not develop respiratory suppression, the difference in RR before treatment between the two groups of patients was more than two per minute (17.5 ± 2.6 versus 15.1 ± 2.3, *P* = 0.00002). This suggests that acoustic RR monitoring might be useful in predicting respiratory suppression. To summarize, as all patients who developed respiratory suppression were detected by acoustic RR monitoring in our study, it seems critical to continuously monitor RR during ESD using the technique to avoid serious respiratory complications.

Our study has some limitations. Firstly, the study was conducted retrospectively in a single institution with a relatively small sample size.Since very few studies are available on the usefulness of acoustic RR monitoring during endoscopic treatment, further well-designed, prospective studies employing large sample size are necessary to verify our findings. Likewise, trials focused on pathological mechanisms underlying respiratory suppression should be undertaken. The second limitation relates to the controversy that surrounds the diagnostic and clinical relevance of respiratory suppression. Developing a standardized definition of respiratory suppression might be essential in order to investigate the effects of sedatives and to delve into the issue of safe and reliable monitoring. Thirdly, acoustic RR monitoring cannot effectively detect signals when the patient is snoring or coughing during endoscopy.

## 5. Conclusions

This is the first study to identify RR before treatment as an independent predictor of respiratory suppression during ESD. Furthermore, our results indicate that acoustic RR monitoring may be more useful than monitoring blood oxygen saturation in patients undergoing upper gastrointestinal ESDs under sedation and may facilitate early detection of respiratory suppression in these patients.

## Figures and Tables

**Table 1:** A comparison of patients who developed respiratory suppression during endoscopic submucosal dissection [RS(+)] and those who did not [RS(−)].

Parameter	RS(−) *n* = 113	RS(+) *n* = 69	*P* value
Gender (male/female)	86/27	42/27	0.03
Age (y, mean ± SD)	69.9 ± 7.6	71.3 ± 9.1	0.20
Height (cm, mean ± SD)	162.6 ± 7.6	159.2 ± 8.2	<0.01
Weight (kg, mean ± SD)	60.2 ± 9.4	56.4 ± 11.3	0.01
Past history (−/+)			
Heart	50/63	38/31	0.16
Lung	101/22	60/9	0.50
Liver	108/5	68/1	0.51
Cr (mg/dL)	1.04 ± 1.0	1.20 ± 1.6	0.60
RR before treatment (mean ± SD)	17.5 ± 2.6	16.5 ± 2.5	0.01
BP < 90 mmHg (−/+)	86/27	49/20	0.45
Dose of sedative (mg) (mean ± SD)	6.5 ± 4.5	6.2 ± 3.4	0.69
Procedure time (min)	105.5 ± 78.1	106.6 ± 50.0	0.15
ESD (esophagus/stomach)	15/98	9/60	0.86

RS: respiratory suppression, RR: respiratory rate, Cr: serum creatinine, and BP: blood pressure.

**Table 2:** Multivariate logistic regression analysis of possible risk factors for respiratory suppression in patients undergoing endoscopic submucosal dissection.

Parameter	OR	OR (95% CI)	*P* value
Gender	1.14	0.44–2.95	0.79
Height	0.96	0.90–1.02	0.16
Weight	0.98	0.94–1.02	0.38
RR before treatment	0.83	0.73–0.95	0.006

RR: respiratory rate, CI: confidence interval.

## Abbreviations

RR: Respiratory rate
ESD: Endoscopic submucosal dissection
OR: Odds ratio
SD: Standard deviation
CI: Confidence interval
Cr: Serum creatinine
BP: Blood pressure.
